# Monitoring Exotic Beetles with Inexpensive Attractants: A Case Study

**DOI:** 10.3390/insects12050462

**Published:** 2021-05-17

**Authors:** Enrico Ruzzier, Andrea Galli, Luciano Bani

**Affiliations:** 1 Department of Agronomy, Food, Natural Resources, Animals and the Environment (DAFNAE), University of Padova, Viale dell’Universita 16, Legnaro, 35020 Padova, Italy; 2 World Biodiversity Association Onlus c/o NAT LAB Forte Inglese, Portoferraio, 57037 Livorno, Italy; luciano.bani@unimib.it; 3 Department of Earth and Environmental Sciences, University of Milano-Bicocca, Piazza della Scienza 1, 20126 Milano, Italy; a.galli57@campus.unimib.it

**Keywords:** alien species, biodiversity, Coleoptera, Nitidulidae, *Popillia*, vinegar

## Abstract

**Simple Summary:**

Detecting and monitoring exotic and invasive beetles is a complex activity, and multiple species still manage to evade controls. Citizen science can be an important adjunct in alien species monitoring programs, but to have a greater chance of success, it must employ traps and attractants that are easy to gather and use. Bottle traps baited with food products are successfully used during long term faunistic surveys, and the same methodology can be adapted to alien species detection and monitoring. In this article, we tested the use of bottles baited with apple cider vinegar, red wine, and 80% ethyl alcohol in capturing exotic and invasive beetles in the surroundings of Malpensa Airport (Italy). The traps proved effective, and in the traps with vinegar as an attractant, they captured four out of five invasive Nitidulidae, as well as the only invasive Scarabaeidae present in the area. *Popillia* *japonica*’s response to apple cider vinegar is documented for the first time and suggests the use of this attractant in monitoring surveys for this species, especially if supported by citizen science programs. The substantial reduction in the activity time of the traps seems to have considerably reduced collateral catches of native fauna.

**Abstract:**

Detecting and monitoring exotic and invasive Coleoptera is a complex activity to implement, and citizen science projects can provide significant contributions to such plans. Bottle traps are successfully used in wildlife surveys and can also be adapted for monitoring alien species; however, a sustainable, large scale trapping plan must take into account the collateral catches of native species and thus minimize its impact on local fauna. In the present paper, we tested the use of bottles baited with standard food products that can be purchased in every supermarket and immediately used (apple cider vinegar, red wine, and 80% ethyl alcohol) in capturing exotic and invasive beetles in the area surrounding Malpensa Airport (Italy). In particular, we reduced the exposition type of the traps in each sampling round to three days in order to minimize native species collecting. We found a significant effect of the environmental covariates (trap placement, temperature, humidity, and forest type) in affecting the efficiency in catching target beetles. Nearly all invasive Nitidulidae and Scarabaeidae known to be present in the area were captured in the traps, with apple cider vinegar usually being the most effective attractant, especially for the invasive *Popillia* *japonica*.

## 1. Introduction

Among European countries, Italy has the most exotic taxa (species that are not native to a specific ecosystem), several of which are invasive (organisms that cause ecological or economic harm in an ecosystem where they are not native) [1]; among these, Coleoptera alone account for more than 300 intercepted or established species [2,3,4,5,6,7,8,9,10,11,12,13]. Insect introductions through human-mediated dispersal is ever increasing because of globalization, and represents a serious threat to biodiversity, local economies and animal and plant health [14]. The ever-increasing number and types of goods transported and the speed at which commercial traffic occurs, associated with the opening of new trade routes, are increasingly resulting in faunal exchanges among and within biogeographic realms [15]. Italy’s predisposition to beetle (Coleoptera) introductions is likely associated with its geographic position in the center of the Mediterranean, being at the crossroads of much commerce to and from Europe [1,16].

Attention and awareness towards alien species have increased in recent decades, even though strategies to reduce future invasions have not yet been implemented in an effective and widespread manner on a global scale [17].

If we consider the number of exotic species recorded in the last few years, and especially those collected fortuitously, it is clear that current monitoring strategies are ineffective at detecting several beetle families. It is for this reason that great effort has been invested in improving monitoring strategies, survey methods, and traps [18,19], with a focus on the main entry points such as seaports and airports (i.e., [20,21]). However, much of these recent developments have targeted primarily families of forest insects, mostly wood borers, such as Curculionidae: Scolytinae and Platypodinae, Cerambycidae, and Buprestidae (e.g., [22,23,24]). Furthermore, biosecurity surveillance suffers from two major issues, namely: (1) effective monitoring strategies, especially those that target multiple taxa, which are generally expensive because of the cost of traps and pheromones, and (2) difficulty in applying targeted monitoring strategies simultaneously and on a national scale.

For this reason, the scientific community is increasingly availing itself of the support of citizen science as a means of strengthening its surveillance capacity (e.g., [25,26]). However, for a monitoring plan supported by citizen science to be effective, it is necessary that it is easily reproducible, low-cost, and does not involve a heavy workload for volunteers.

Bottle traps is a methodology commonly and successfully used in faunistic surveys [27,28]. Bottle traps are inexpensive, easy to make and transport, and have been recently suggested and applied in bark and ambrosia beetle (Scolytinae and Platypodinae) monitoring through citizen participation [29,30,31]. 

Based on these concepts, we decided to conduct a trial intended to evaluate if bottle traps could be used to monitor certain exotic beetles that are not commonly targeted with standard traps (e.g., Lindgren funnels traps and cross-vane panel traps) and pheromones, especially using food products as lures that can be purchased directly at the supermarket. In the scientific literature, there are many attractive mixtures made from fermented goods (such as honey, bananas, and beer; e.g., [32,33]). However, as we could not expect all volunteers to produce such attractive mixtures, we opted for three affordable products that can be used directly as they are when purchased, namely: food-grade ethyl alcohol (which will be referred to as alcohol), red wine, and apple cider vinegar.

However, as the objective of this trial was to target exotic species, the exposure times of our traps were reduced to only three days each during each survey round. This decision started from the assumption that invasive species are characterized by biological and ecological traits that make them more prone to rapidly respond to generic olfactory stimuli (e.g., food/reproduction sources) compared with native species. Given the collateral catch that these monitoring activities can have on native species, we expected that reducing the traps’ exposure time would maintain the capture of exotics, while minimizing the impact on native species.

In the present study, we evaluated (1) the effectiveness of bottle traps baited with non-fermented goods and short exposure times in catching exotic beetles, (2) the beetle families and species collected in terms of diversity and abundance, and (3) how the different baits varied in attractiveness to target beetle species. Furthermore, we tried to assess the effect of temperature, humidity, environmental surroundings, and trap placement on trapping efficiency.

## 2. Materials and Methods

### 2.1. Study Area

**Figure 1:**
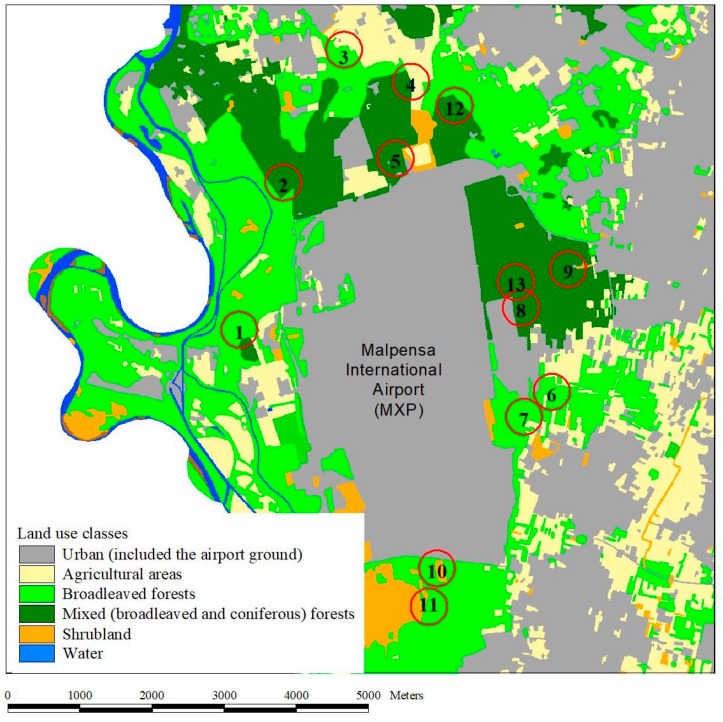
Study area. Map illustrating land use classes and the sampling localities in the surrounding of the Malpensa International Airport, MXP (Ferno and Somma Lombardo municipalities, Varese Province, Italy); numbers indicate the 13 sampling sites where the trial was performed.

The study area is centered on the Malpensa International Airport (MXP), the largest international airport in northern Italy, located in the Ferno Municipality (Varese Province), within the Lombardy Regional Park of Ticino Valley, about 50 km northwest of Milan. The airport grounds are largely surrounded by deciduous and mixed (broad-leaved and coniferous) forests and urban areas, and, to a lesser extent, agricultural areas and shrubland (Figure 1). Among the trees, the dominant taxa are oaks (*Quercus* spp.), maples (*Acer* spp.), and Scots pine (*Pinus sylvestris*). Most of the woodlands are managed as coppice, and the presence of exotic trees is not negligible, with the Locust tree (*Robinia pseudoacacia*) among the most widespread species.

### 2.2. Experimental Design, Traps, and Baits

The traps were placed in 13 sampling sites identified to account for habitat covariates, i.e., the forest type (broadleaved vs. mixed) and forest edge vs. forest interior condition (interior: condition with forest fractional cover >90% evaluated in a buffer of 250 m around trapping site), based on DUSAF digital cartography [34] (Table 1). In each sampling site, three traps were used, each baited with either 80% food alcohol, commercial red wine (11.5% alcohol), and apple cider vinegar (4% acetic acid), for a total of 39 traps. No preservatives were added. In each sampling site, the three traps were placed along the perimeter of an ideal circle with about a 15 m radius (thus having a distance of at least 30 m among traps) to avoid their mutual influence on capture events; all traps were placed 2.5 m from the ground. Each trap was built using a 500 mL volume PET (polyethylene terephthalate). A 5 × 8.5 cm window was created on half of each bottle by removing a sizeable lateral portion, leaving the rest of the bottle intact (to guarantee the structural integrity and support of the modest weight of the bait). Each set of baited traps was left in action for 72 h, and then removed and emptied each week between 31 July and 29 September 2020, for a total of seven trapping periods. For each trapping session, we recorded the absolute minimum and maximum temperature during the three days of activity of the traps, the three-days mean of the mean daily temperatures, and the three-days mean of the humidity degree. As the three temperature values were correlated within each trapping session, we used the absolute maximum temperature (Table 2). The meteorological data were obtained from the airport meteorological station.

All of the non-Coleoptera (i.e., Diptera, Lepidoptera, Hymenoptera, and Hemiptera) collected during the survey were not considered in the analyses and were discarded.

### 2.3. Analyses

We evaluated the overall effectiveness of each type of bait (red wine, vinegar, or alcohol), considering the overall number of individuals of exotic species pooled together caught by each kind of bait in each site and session, accounting for both habitat and meteorological covariates. The same model was applied to the overall number of individuals for the native species subset. The analysis was performed using a negative binomial regression for modelling count data using *MASS* package [35] in R version 4.0.3 [36]. The assessment of the data distribution for the dependent variable was performed using the *fitdistrplus* package [37]. Plots of the conditional effects (habitat variables) and main effect (meteorological variables) of covariates were made using the *sjPlot* package [38], whose functionality depends on the *ggplot2* package [39]. The same analysis was then performed to evaluate the specific overall effectiveness of each bait, considering the number of individuals pertaining to the most commonly trapped species.

## 3. Results

**Figure 2:**
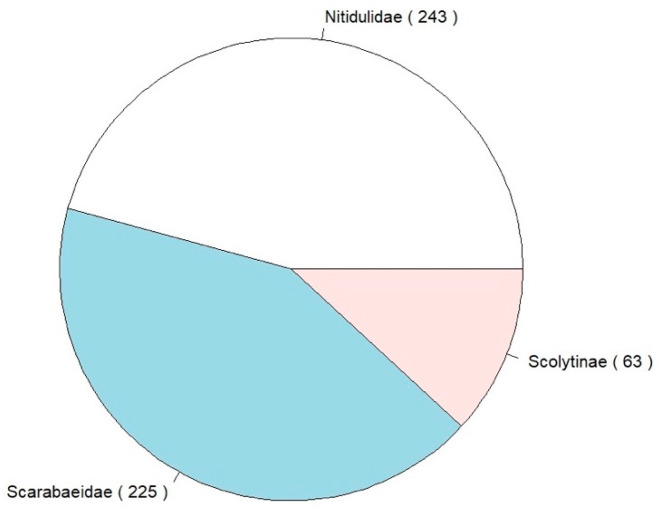
Beetle families collected during the survey; total number of individuals given in brackets.

**Figure 3:**
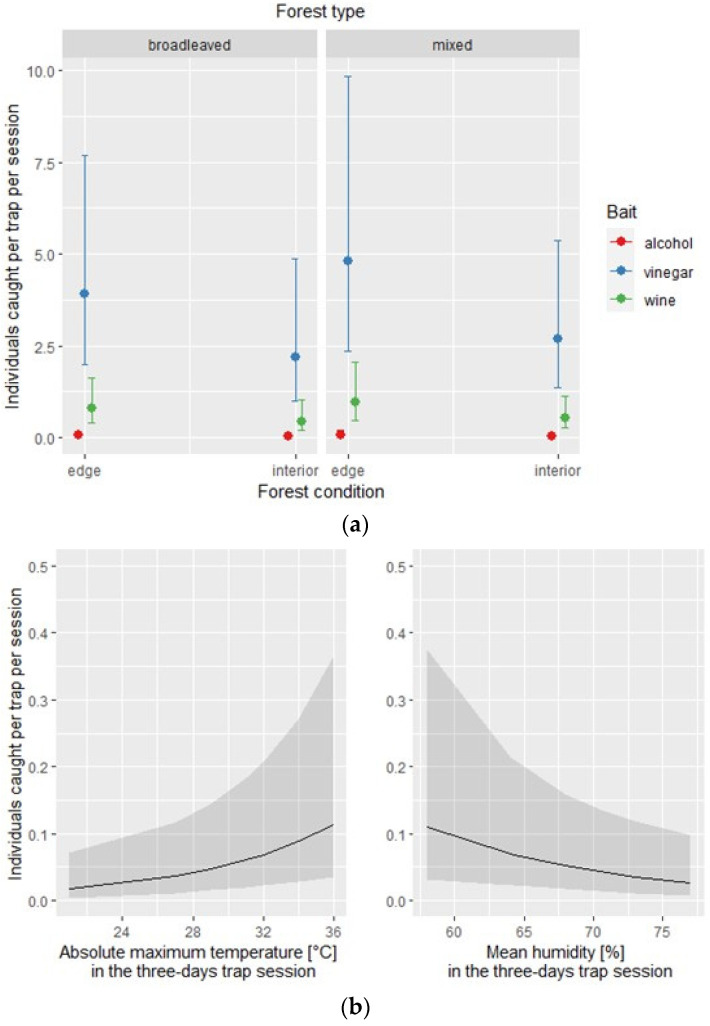
Exotic species: effect of the covariates of the negative binomial regression model affecting the number of individuals caught by each of the three traps per site and per session; (**a**) conditional effect of habitat covariates (forest type: broadleaved vs. mixed; forest condition: edge vs. interior); (**b**) main effect of meteorological covariates.

During the two-months survey period (i.e., seven three-day sessions), in the 13 sampling sites, we caught a total of 531 individuals (437 pertaining to exotic and 94 to native species). The 14 species of beetles collected belonged to Scolytinae, Nitidulidae, and Scarabaeidae (Figure 2 and Table 3). Among the species collected, five are considered invasive in Europe (*Carpophilus lugubris*, *Epuraea luteola*, *Epuraea ocularis*, *Glischrochilus quadrisignatus*, and *Popilia japonica*; Figure 3).

The most common species caught were *P*. *japonica* (Scarabaeidae: Rutelinae), with 212 individuals (40% of the overall individual caught and 49% of the individuals of exotic species), and *E*. *ocularis* (Nitidulidae), with 159 individuals (30% of the overall individual caught and 36% of the exotic species). The third most common species was the native *X*. *saxesenii* (Scolytinae), with 58 individuals (11% of the overall individual caught and 62% of native species).

The negative binomial regression models used to assess each bait’s effectiveness in attracting the overall number of exotic and native species explained 40% and 13% of the sample deviance, respectively. For the exotic species subset, the model identified the type of bait, the absolute maximum temperature, and the mean humidity as statistically significant for affecting the overall number of individuals caught in each of the three traps per site and session (Table 4). The conditional effect of each covariate is depicted in Figure 3.

**Figure 4:**
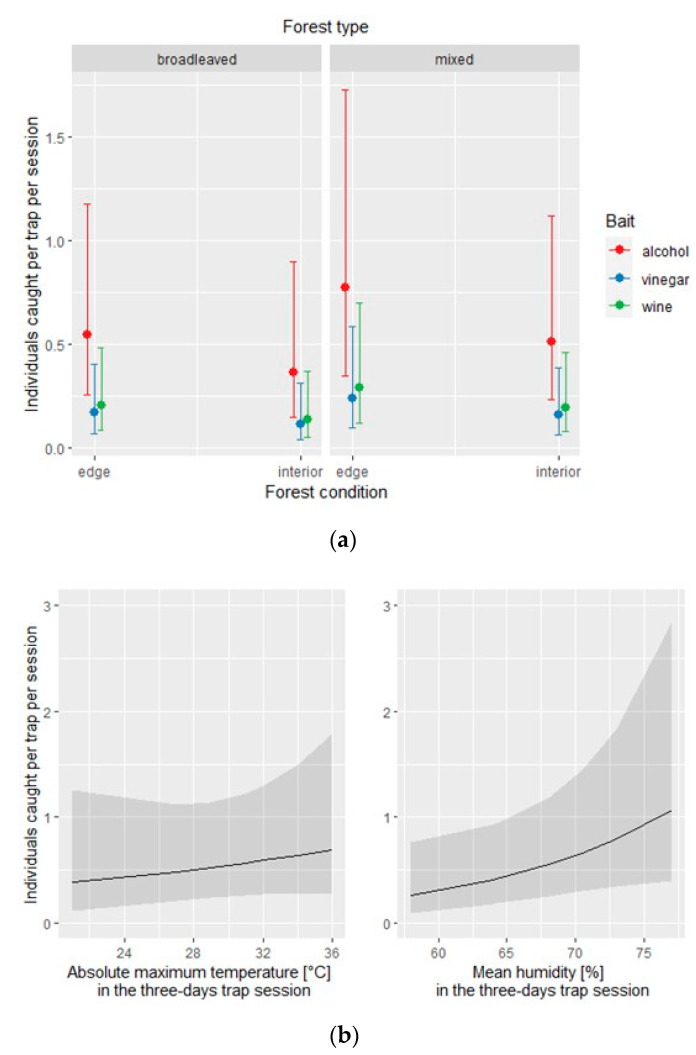
Native species: effect of the covariates of the negative binomial regression model affecting the number of individuals caught by each of the three traps per site and per session; (**a**) conditional effect of habitat covariates (forest type: broadleaved vs. mixed; forest condition: edge vs. interior); (**b**) main effect of meteorological covariates.

The same model applied to the native species subset identified the type of bait and the mean humidity as statistically significant (Table 5). The conditional effect of each covariate is depicted in Figure 4.

For the three most common trapped species (two exotic and one native), the models for the *P*. *japonica*, *E*. *oculari*, and *X*. *saxesenii* explained 72%, 40%, and 32% of the sample deviance, respectively. We must highlight that all of the individuals, but one of *X*. *saxesenii,* were caught in alcohol. Thus, to find the covariate that affects the number of individuals caught, we used only those collected by the traps triggered by alcohol. 

**Figure 5:**
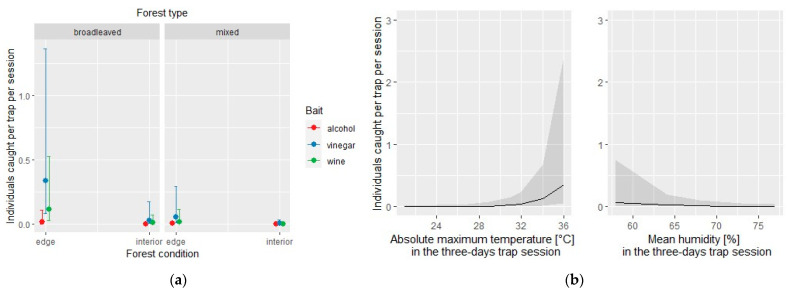
*Popillia japonica*: effect of the covariates of the negative binomial regression model affecting the number of individuals caught by each of the three traps per site and per session; (**a**) conditional effect of habitat covariates (forest type: broadleaved vs. mixed; forest condition: edge vs. interior); (**b**) main effect of meteorological covariates.

For *P*. *japonica*, the type of bait’s effectiveness differed significantly, and both the habitat and meteorological covariates significantly affected the number of individuals caught. Specifically, both vinegar and wine positively enhanced the trap efficiency in collecting *P*. *japonica*, with vinegar being more effective than wine. Furthermore, the number of collected specimens increased with increasing temperatures and captures, and were substantially higher when traps were placed at the edges of broadleaved forests (Table 6). The conditional effect of each covariate is depicted in Figure 5.

**Figure 6:**
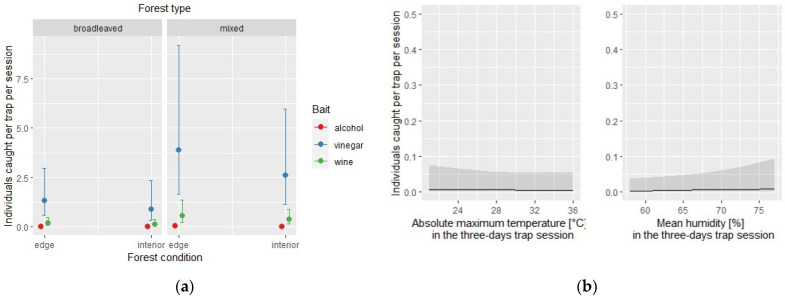
*Epuraea ocularis*: effect of the covariates of the negative binomial regression model affecting the number of individuals caught by each of the three traps per site and per session; (**a**) conditional effect of habitat covariates (forest type: broadleaved vs. mixed; forest condition: edge vs. interior); (**b**) main effect of meteorological covariates.

For *E*. *ocularis*, the type of bait also significantly influenced the effectiveness of trapping, but only the habitat covariates showed a substantial effect on the number of individuals caught, and particularly the forest type. For this species, vinegar and wine positively enhanced the trap efficiency, with vinegar also being more efficient than wine in this case; in addition, mixed forest enhanced the traps’ efficiency (Table 7). The conditional effect of each covariate is depicted in Figure 6.

**Figure 7:**
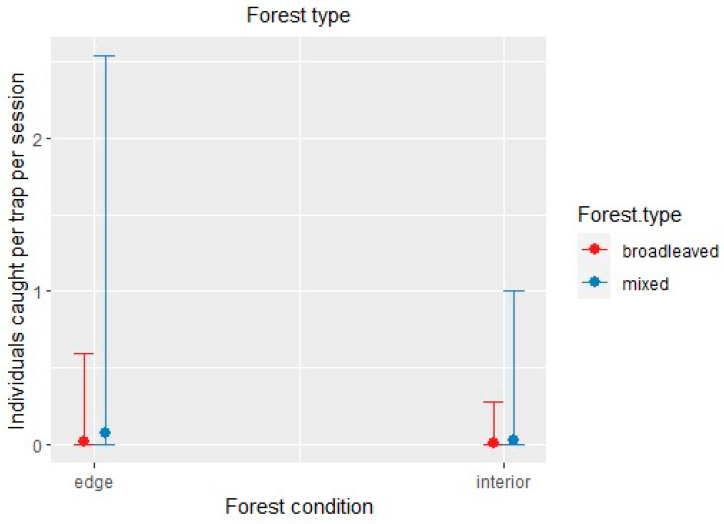
*Xyleborinus saxesenii*: effect of the negative binomial regression model’s covariates affecting the number of individuals caught by the trap triggered with alcohol per site and session. Only the conditional effect of habitat covariates (forest type: broadleaved vs. mixed; forest condition: edge vs. interior) is shown, as neither of the two meteorological variables showed significant effects.

For *X*. *saxesenii*, only the bait type was significant in determining the number of individuals caught in the traps Table 8. The conditional effect of covariates is depicted in Figure 7.

## 4. Discussion

The survey allowed for the collection of thirteen different species among Scolytinae, Nitidulidae, and Scarabaeidae. It is striking that no other beetle family responded positively to the attractants, suggesting that neither the type of traps nor the attractants were adequate; it is also plausible that the sampling season was too late compared with most of the species’ phenology. If we exclude Scolytinae, represented by two native species which were almost exclusively collected with alcohol baited traps, both Nitidulidae and Scarabaeidae were included as invasive species. Four out of the eight species of Nitidulidae belong to invasive taxa, representing the 90% of the total number of the nitidulid specimens. *Carpophilus lugubris* is one of the ten *Carpophilus* species indicated as invasive in Italy [40]; however, it most probably is the only species present in the area, given its recent introduction and expansion throughout Veneto and Friuli Venezia Giulia [41,42]. *Epuraea luteola* and *Epuraea guttata* are two of the three invasive *Epuraea* occurring in Italy, but the only occur in Lombardy, as the third species is limited to southern Italy [40]. *Glischrochilus quadrisignatus* is the only invasive species of the genus in Italy [40]. *Stelidota geminata* (Say, 1825), the only other invasive nitidulid present in Lombardy [43], was not recorded in our survey. *Popillia japonica* is the only invasive Scarabaeidae in Italy [16]; this species alone constituted about 97% of the scarabaeoid specimens.

The fact that this simple trial was able to detect almost, if not all, of the exotic Nitidulidae and Scarabaeidae present in the area suggests, at least for these two families, how home-made traps baited with food products, using apple cider vinegar in particular, may be effective at detecting exotic and invasive beetles, and could be an important addition to monitoring plans around entry points. Long standing food-baited traps are very effective in catching beetles and, in several cases, they have to be modified in order not to kill rare and threatened species (e.g., [44,45]). The proportion of exotic compared with native species collected in our trial suggests that minimizing the activity period of the traps does not substantially affect the traps’ capacity in catching non-native species. Trapping efficiency against exotic species seems to be substantially affected by trap placement (margin vs. interior of forest), absolute maximum temperature, and mean humidity; however, this result may be substantially biased by the two overrepresented *E*. *ocularis* and *P*. *japonica*. A similar effect may be caused by *X*. *saxesenii* on the native species pool. As expected, Scolytinae was the only group responding systematically to alcohol traps, and the fact that the native *X*. *saxesenii* constituted almost all of the individuals collected is perfectly in line with the tendencies of this species to respond to a wide range of ethanol concentrations [46]. Furthermore, the efficiency when catching *X*. *saxesenii* is not affected by the environmental covariates, and is most probably attributable to the species dispersal capabilities, long phenology, and polyphagy.

Nitidulidae is a group of primary interest, given the number of exotic and invasive species introduced in Europe [40]. The effectiveness of vinegar traps in rapidly detecting exotic sap beetles may serve as an important tool in monitoring new introductions or the spread of newly acclimatized species. Fermented baits are commonly used to investigate Nitidulidae [47,48]; the capability of *E*. *luteola*, *E*. *ocularis*, *C*. *lugubris,* and *G*. *quadrisignatus* to rapidly respond to wine and vinegar seems promising in using bottle traps for their capture; furthermore, given the similar ecological niche that these have with most of the others invasive species, we may expect a similar attraction efficiency by both attractants. In addition to direct damage to crops, Nitidulidae can be vectors of important pathogenic fungi such as Ophiostomatales [49] and Microascales [50]; consequently, their early detection may have a relevant role as phytosanitary security. The substantial effect given by the forest type in the capture efficiency of *E*. *ocularis* is probably attributable to its generalist habits and polyphagy, with adults able to feed on rotten fruit, flowers, sapping trees, and larvae developing in the fruit body of tree-fungi or decaying organic matter [40,51].

*Popillia japonica* is a highly polyphagous invasive pest outside its native range, so far limited to Lombardy and Piedmont in Italy [52]. Because of the substantial damage it is able to cause, as well as its excellent dispersal capacity, this species is currently controlled through mass trapping, with traps commonly baited with chemical attractants such as food-type volatile and sex pheromones [53,54,55]. The capture of this species with both wine and vinegar traps was unexpected and constituted an absolute novelty, as it is the first response of *P*. *japonica* towards these two attractants, vinegar in particular. Scarabaeidae Rutelinae are rarely collected with fruit/fermented and baited traps in the Palearctic [56,57], while they constitute a substantial fraction of the tropics’ trapped biomass [58]. However, a particular sensitivity of *P*. *japonica* towards volatile compounds released during fruit ripening or rotting can be deduced from Hammons et al. [59,60], where the species have been repeatedly documented as feeding on grapes in the USA. Concerning environmental covariates, the trapping efficiency against *P*. *japonica* is substantially affected by forest type, forest cover condition, and absolute maximum temperature. Forest composition and trap placement (forest edge/interior) have a significant effect, as *P*. *japonica* prefers ecotones, areas hosting the greater variety of feeding plants, and where the species move by flying. Furthermore, ecotonal areas may present grass patches suitable for egg deposition and larval development [61]. The positive effect of high temperatures in increasing trapping efficiency is defined by a general increase of *P*. *japonica* activity combined with a greater attractant volatility [62].

Vinegar efficiency against Nitidulidae and *P*. *japonica* in heterogeneous habitats suggests a good effectiveness of the traps in agricultural and peri-urban environments, as well as other anthropized entry points such as airports and ports. Furthermore, adopting short trapping sections repeated over time seems to not affect native beetles through an unnecessary over-trapping.

## 5. Conclusions

The traps used in this study can be produced in a short time, using recycled or easily available materials. Furthermore, as the traps are light and compact, they can be comfortably managed by one person, even in large numbers. The attractants are readily available at a low cost, making this survey technique very useful in citizen science projects for large-scale monitoring projects. Apple cider vinegar has proven to be the most affordable and still the most efficient bait, capable of attracting exotic and invasive beetles in almost all conditions; wine has intermediate attractivity and may be used as a coadjutant of vinegar; conversely, in our context, high-grade alcohol targets exclusively native Scolytinae. As what has been presented here is only a first trial, it will certainly be interesting to replicate the monitoring using different types of vinegar or wine and to evaluate the influence of other potential co-factors, including seasonality. It would be interesting to identify and test new types of inexpensive attractants for other families of beetles other than those used in this study.

As citizen science data are not usually collected following a sampling design typical of standardized monitoring programs, we stress that the collection of environmental variables, beside the collection of the specimens, is crucial for a correct interpretation of the records. Specifically, the position of the traps is essential information that must be provided by citizens involved in monitoring activities, along with the baits used and the time of exposure of the traps. In particular, if these monitoring projects are directed against invasive species, citizen scientists should keep in mind that the time exposure of baited traps should be minimized in order to reduce the impact on native fauna. 

Given the strong attractivity of vinegar against *P*. *japonica*, it is plausible that the massive trapping of this species through a supervised citizen science action may become a substantial contribution and integration to the control strategies developed by local phytosanitary institutions, especially in cultivated and suburban areas.

## Figures and Tables

**Table 1:** Location of the sampling sites in which the group of three traps was triggered with different baits. The forest covers in a buffer of 250 m around the sampling site used to define the forest conditions (interior condition: forest cover >90%) and forest type.

Site Number	Nord (WGS84/UTM Zone 32N)	East (WGS84/UTM Zone 32N)	Forest Cover (250-m Buffer)	Forest Condition	Forest Type
1	5,053,409	476,170	98%	interior	broadleaved
2	5,055,451	476,779	100%	interior	mixed
3	5,057,292	477,621	58%	edge	broadleaved
4	5,056,847	478,549	52%	edge	mixed
5	5,055,783	478,335	87%	edge	mixed
6	5,052,549	480,498	79%	edge	broadleaved
7	5,052,204	480,113	88%	edge	broadleaved
8	5,053,714	480,070	76%	edge	mixed
9	5,054,249	480,724	99%	interior	mixed
10	5,050,105	478,906	82%	edge	broadleaved
11	5,049,577	478,796	100%	interior	broadleaved
12	5,056,516	479,151	100%	interior	mixed
13	5,054,071	479,998	99%	interior	mixed

**Table 2:** Meteorological data obtained from the airport station for each of the three-days trapping sessions.

Session Number	Placement Date	Control Date	Abs. Min. Temp. (°C) 3-Day Session	Abs. Max. Temp. (°C) 3-Day Session	Mean Temp. (°C) 3-Day Session	Mean Humidity3-Days Session
1	31 July 2020	3 August 2020	18	36	26.3	64.0
2	7 August 2020	10 August 2020	17	34	26.5	58.0
3	14 August 2020	17 August 2020	18	32	24.5	68.0
4	28 August 2020	31 August 2020	13	27	21.0	77.0
5	4 September 2020	7 September 2020	14	29	20.8	73.0
6	11 September 2020	14 September 2020	17	31	23.3	70.5
7	25 September 2020	28 September 2020	4	21	13.0	64.3

**Table 3:** Coleoptera species collected during the survey. (*V*—Vinegar; *W*—wine; *E*—Alcohol.)

Species	Family/Subfamily	Origin	Status	Attractant	N° of Individuals
*Carpophilus lugubris* Murray 1864	Nitidulidae	Nearctic	Invasive	*V*(3); *W*(2)	5
*Cryptarcha strigata* (Fabricius, 1787)	Nitidulidae	W-Palaearctic	Native	*W*(1)	1
*Epuraea guttata* (Olivier, 1811)	Nitidulidae	W-Palaearctic	Native	*V*(6); *W*(1)	7
*Epuraea luteola* (Erichson, 1843)	Nitidulidae	E-Palaearctic	Invasive	*V*(5)	5
*Epuraea ocularis* (Fairmaire, 1849)	Nitidulidae	E-Palaearctic	Invasive	*V*(176); *W*(28); *E*(1)	205
*Epuraea unicolor* (Olivier, 1790)	Nitidulidae	Palaearctic	Native	*V*(5)	5
*Glischrochilus quadrisignatus* (Say, 1835)	Nitidulidae	Nearctic	Invasive	*V*(3); *W*(1)	4
*Soronia grisea* (Linnaeus, 1758)	Nitidulidae	Palaearctic	Native	*V*(2); *W*(9)	11
*Anisandrus dispar* Fabricius, 1792	Scolytinae	Palaearctic	Native	*E*(4)	4
*Xyleborinus saxesenii* (Ratzeburg, 1837)	Scolytinae	Palaearctic	Native	*W*(1) *E*(581)	59
*Cetonia aurata* (Linnaeus, 1758)	Scarabaeidae	W-Palaearctic	Native	*W*(1)	1
*Popillia japonica* (Newman, 1838)	Scarabaeidae	E-Palaearctic	Invasive	*V*(161); *W*(51); *E*(6)	218
*Potosia cuprea* (Fabricius, 1775)	Scarabaeidae	W-Palaearctic	Native	*W*(1)	1
*Protaetia speciosa* (Adams, 1817)	Scarabaeidae	W-Palaearctic	Native	*W*(1)	5

**Table 4:** Exotic species. Effectiveness of baits in attracting individuals of all species pooled together, accounting for the effects of habitat and meteorological covariates considered in the negative binomial regression model.

Covariates	Estimate	SE of Estimate	*z*-Value	*p*
(intercept)	−1.657	2.998	−0.553	0.581
Bait: vinegar	4.311	0.584	7.379	<0.001
Bait: wine	2.705	0.594	4.552	<0.001
Forest type: mixed forests	0.201	0.367	0.549	0.583
Forest cover condition: interior	−0.579	0.371	−1.559	0.119
Absolute maximum temperature	0.127	0.044	2.901	0.004
Mean humidity	−0.075	0.033	−2.296	0.022

**Table 5:** Native species. Effectiveness of baits in attracting individuals of all species pooled together, accounting for the effects of habitat and meteorological covariates considered in the negative binomial regression model.

Covariates	Estimate	SE of Estimate	*z*-Value	*p*
(intercept)	−6.772	3.344	−2.025	0.042
Bait: vinegar	−1. 187	0.479	−2.479	0.013
Bait: wine	−0.986	0.467	−2.110	0.035
Forest type: mixed forests	0.345	0.403	0.857	0.391
Forest cover condition: interior	−0.411	0.406	−1.012	0.312
Absolute maximum temperature	0.039	0.050	0.786	0.431
Mean humidity	0.073	0.036	2.038	0.042

**Table 6:** *Popillia japonica*. Effectiveness of baits in attracting individuals of the species, accounting for the effects of habitat and meteorological covariates considered in the negative binomial regression model.

Covariates	Estimate	SE of Estimate	*z*-Value	*p*
(intercept)	−9.751	9.453	−1.032	0.302
Bait: vinegar	3.136	0.922	3.401	0.001
Bait: wine	2.047	0.940	2.178	0.029
Forest type: mixed forests	−1.827	0.680	−2.685	0.007
Forest cover condition: interior	−2.574	0.795	−3.237	0.001
Absolute maximum temperature	0.519	0.172	3.012	0.003
Mean humidity	−0.147	0.078	−1.893	0.058

**Table 7:** *Epuraea ocularis*. Effectiveness of baits in attracting individuals of the species, accounting for the effects of habitat and meteorological covariates considered in the negative binomial regression model.

Covariates	Estimate	SE of Estimate	*z*-Value	*p*
(intercept)	−8.111	3.705	−2.189	0.029
Bait: vinegar	5.382	1.115	4.829	<0.001
Bait: wine	3.410	1.129	3.019	0.003
Forest type: mixed forests	1.083	0.466	2.327	0.020
Forest cover condition: interior	−0.392	0.461	−0.851	0.395
Absolute maximum temperature	−0.016	0.052	−0.311	0.756
Mean humidity	0.051	0.040	1.292	0.196

**Table 8:** *Xyleborinus saxesenii*. Effectiveness of alcohol in attracting individuals of the species, accounting for the effects of habitat and meteorological covariates considered in the negative binomial regression model.

Covariates	Estimate	SE of Estimate	*z*-Value	*p*
(intercept)	−64.815	45.690	−1.419	0.156
Forest type: mixed forests	1.544	0.860	1.796	0.073
Forest cover condition: interior	−0.972	0.861	−1.129	0.259
Absolute maximum temperature	0.711	0.596	1.194	0.233
Mean humidity	0.597	0.390	1.530	0.126

## Data Availability

The original dataset is available under request.
